# Approaches to Measuring Beta Cell Reserve and Defining Partial Clinical Remission in Paediatric Type 1 Diabetes

**DOI:** 10.3390/children11020186

**Published:** 2024-02-02

**Authors:** Elaine C. Kennedy, Colin P. Hawkes

**Affiliations:** 1 Department of Paediatrics and Child Health, University College Cork, T12 DC4A Cork, Ireland; 2 INFANT Research Centre, University College Cork, T12 DC4A Cork, Ireland; 3 Perelman School of Medicine, University of Pennsylvania, Philadelphia, PA 19104, USA; 4 Division of Endocrinology and Diabetes, Children’s Hospital of Philadelphia, Philadelphia, PA 19104, USA

**Keywords:** diabetes, c-peptide, honeymoon, beta cell, residual, teplizumab, remission

## Abstract

Context: Type 1 diabetes (T1D) results from the autoimmune T-cell mediated destruction of pancreatic beta cells leading to insufficient insulin secretion. At the time of diagnosis of T1D, there is residual beta cell function that declines over the subsequent months to years. Recent interventions have been approved to preserve beta cell function in evolving T1D. Objective: The aim of this review is to summarise the approaches used to assess residual beta cell function in evolving T1D, and to highlight potential future directions. Methods: Studies including subjects aged 0 to 18 years were included in this review. The following search terms were used; “(type 1 diabetes) and (partial remission)” and “(type 1 diabetes) and (honeymoon)”. References of included studies were reviewed to determine if additional relevant studies were eligible. Results: There are numerous approaches to quantifying beta cell reserve in evolving T1D. These include c-peptide measurement after a mixed meal or glucagon stimuli, fasting c-peptide, the urinary c-peptide/creatinine ratio, insulin dose-adjusted haemoglobin A1c, and other clinical models to estimate beta cell function. Other biomarkers may have a role, including the proinsulin/c-peptide ratio, cytokines, and microRNA. Studies using thresholds to determine if residual beta cell function is present often differ in values used to define remission. Conclusions: As interventions are approved to preserve beta cell function, it will become increasingly necessary to quantify residual beta cell function in research and clinical contexts. In this report, we have highlighted the strengths and limitations of the current approaches.

## 1. Background

Type 1 diabetes (T1D) results from the autoimmune T-cell-mediated destruction of pancreatic beta cells leading to insufficient insulin secretion. Four stages of clinical progression have been described as the beta cell reserves deplete. There is normal glucose tolerance at the first stage, with subsequent increasing dysglycemia and an absence of endogenous insulin secretion at the final stage [1].

At the time of diagnosis of T1D, there is residual beta cell function that declines over the subsequent months to years [2,3], with significant variation in trajectory between patients [4,5]. Four years after diagnosis of T1D, approximately 30% of patients no longer have detectable beta cell reserve, compared with 2% at the end of the first year [6]. These residual functional pancreatic beta cells continue to produce endogenous insulin, reducing the requirement for exogenous insulin administration. For some patients, beta cell decline is incomplete, and a low level of insulin secretion is maintained throughout life. In the short-term, residual beta cell function is associated with a reduced risk of hypoglycaemia [7], and in the longer term, reduced rates of T1D complications are seen [8,9,10].

Many interventions to delay disease progression have been studied and teplizumab, a humanised anti-CD3 monoclonal antibody, has recently been approved as a disease-modifying medication that can slow the development of T1D. This is approved in individuals aged 8 years or older with two or more detectable diabetes-related autoantibodies, as well as evidence of preclinical hyperglycaemia [11,12]. Recent studies also suggest some preservation of beta cell function when teplizumab is used in patients with newly diagnosed T1D [13,14]. However, clinical outcomes including glycaemic control and insulin doses were not impacted by teplizumab treatment in this population [13].

Given the availability of beta cell-preserving interventions in T1D, the ability to accurately assess beta cell function is critical to assess treatment efficacy. Various approaches to quantifying beta cell function have been described clinically and in research, including stimulated and single c-peptide measurement, as well as mathematical models including insulin-dose-adjusted glycosylated haemoglobin A1c (HbA1c) estimation. The aim of this review is to summarise the approaches used to assess residual beta cell function in evolving T1D, and to highlight potential future directions.

## 2. Search Methodology

A PubMed search was performed on 3/1/23. The terms “(type 1 diabetes) and (partial remission)” and “(type 1 diabetes) and (honeymoon)” were included. Studies were limited to those involving individuals aged 0–18 years. References of included articles were reviewed and relevant articles were also included. Therefore, some studies relating to an adult population may have been included if deemed to be relevant to the paediatric literature by the authors. In each study, the methodology of assessment and definition of partial remission were extracted. In studies where new approaches were validated successfully against prior methodologies, both definitions were included. Where novel approaches were not validated, they were summarised separately.

## 3. Results

A total of 145 studies were identified by our search criteria, and 90 of these were included in our review. Examples of exclusion criteria include if the manuscript did not define partial clinical remission, or if we were unable to access the full paper and the definition of remission was not included in the abstract. The included studies describe 7 different methodologies for defining clinical partial remission. In studies using the same methods to assess for residual function, the threshold used to define remission often varied (Table 1).

## 4. Approach 1: C-peptide Measurement

Directly measuring circulating insulin levels to estimate beta cell reserve in T1D is not feasible for a number of reasons. There can be assay cross-reactivity between endogenous and exogenous insulin [101], endogenous insulin has a short half-life [102] and up to 80% of secreted insulin is excreted through hepatic first-pass metabolism [101,103,104,105]. Insulin and c-peptide are secreted in an equimolar ratio by the pancreatic beta cells following cleavage of proinsulin. Circulating c-peptide levels subsequently reflect insulin production [103].

C-peptide has physiological properties that make it a more suitable marker of beta cell reserve than insulin. C-peptide has a longer half-life, undergoes negligible hepatic clearance, and is not detected by conventional insulin assays, thereby permitting its use in patients using exogenous insulin [106,107]. C-peptide can be measured in both serum and urine [106,108,109,110]. Although some newer assays use monoclonal antibodies and report improved reproducibility, multiple c-peptide assays are available, and this can affect the comparability of results [106]. Serum samples can be fasting, or stimulated [106], and both approaches have been used to estimate beta cell reserve.

### 4.1. 1a. Stimulated C-peptide

C-peptide levels measured after a stimulus helps to directly quantify beta cell reserve. Compared with fasting c-peptide measurements, stimulated tests elicit measurable c-peptide responses even in those who have undetectable fasting c-peptide concentrations [111]. Both MMTT and glucagon stimulation tests have demonstrated reproducibility [111]. MMTT is widely used in clinical trials to estimate beta cell stimulation and involves an overnight fast followed by IV cannulation, ingestion of a standardised liquid meal and repeated phlebotomy over a two to four hour period [84,111,112,113,114,115]. Peak response usually occurs at 90 min, and c-peptide levels return to baseline at 120 min. The AUC c-peptide or peak stimulated c-peptide measured during an MMTT are generally considered the preferred methods for measuring beta cell function in interventional trials related to T1D [110,112,116]. In clinical settings, it is not a practical method of regularly measuring beta cell reserve, especially in the paediatric population, where venepuncture can be more technically challenging and associated with distress for the child [110].

Intravenous glucagon is an alternative stimulus for c-peptide secretion to the mixed meal. Following glucagon administration, c-peptide is measured over the following ten minutes. Both the mixed meal and glucagon have reproducible results when performed in the same patient 3 to 10 days apart, but the MMTT is more reproducible. Furthermore, peak c-peptide levels are higher following the MMTT [111]. Compared to the MMTT, patients described more nausea with the glucagon stimulation test, but the shorter duration of the study may be advantageous [111].

Single c-peptide measurement at 90 min following an MMTT (90CP) strongly correlates with AUC c-peptide [110]. From a practical standpoint, this c-peptide measurement at 90 min still requires the same pre-MMTT preparation and a longer test time for the patient compared with a fasting c-peptide sample. However, it requires fewer blood samples, thereby reducing cost and test duration.

### 4.2. 1b. Fasting C-peptide

Fasting c-peptide levels correlate with other measures of beta cell reserve, including area under the curve (AUC) c-peptide, following an MMTT [110]. Fasting c-peptide, corrected for fasting glucose, has also been shown to correlate strongly with AUC c-peptide. This has been suggested as a practical alternative to AUC c-peptide measured during a MMTT, or to a fasting c-peptide measurement on its own (R^2^ 0.94 vs. 0.88) [112]. However, the sensitivity of fasting c-peptide measurement for residual beta cell function is lower than the MMTT, as many children with undetectable fasting c-peptide levels will have measurable stimulated c-peptide [111].

### 4.3. 1c. Urinary C-peptide/Creatinine Ratio

The urinary c-peptide-to-creatinine ratio (UCCR) has been used as an alternative to stimulated serum c-peptide measurement to assess residual beta cell function. Urinary measurement 120 min into an MMTT correlates well with the 90 min serum c-peptide concentrations (r = 0.97). Similarly, UCCR measured at home after an evening meal also showed correlation with the 90 min serum c-peptide (r = 0.91) [108]. However, fasting UCCR does not correlate well with AUC c-peptide measured during an MMTT (r = 0.4172) [109]. These results suggest that at-home urinary c-peptide measurements could be used to quantify beta cell function without the time and cost associated with stimulated c-peptide tests, but correlate more closely with stimulated c-peptide levels when measured in a postprandial rather than a fasting state.

## 5. Approach 2: Clinical Models

The use of stimulation testing to estimate beta cell function is generally confined to clinical research and not routinely used in clinical care. Clinical models that estimate beta cell reserve without the use of stimulated tests have been used as surrogate markers of endogenous insulin secretion. Various components have been suggested as useful contributors to these clinical models.

### 5.1. 2a Insulin Dose-Adjusted A1C

Exogenous insulin requirements increase as T1D progresses and beta cell reserve diminishes. Total daily dose of insulin (TDD) has been used as a surrogate marker of beta cell function, but TDD needs to account for glycaemic control if this measure is to be used. TDD is often used in conjunction with HbA1c as a means of estimating if a patient is in ‘honeymoon’ or not (Table 1) [22,24,26,117,118]. HbA1c is limited in its ability to estimate beta cell function in the context of evolving deficiency and it does not represent real-time beta cell function, rather an estimate of function over the previous three months [3]. Consequently, variation in HbA1c-adjusted insulin requirement values may not be as reflective of endogenous insulin production as measured through stimulated c-peptide tests [119].

Insulin dose-adjusted A1C (IDAA1C) [5] is the most commonly used clinical method to estimate residual beta cell function at present (Table 1). This model uses current insulin TDD and HbA1c levels to predict if a patient is in remission, as defined by a stimulated c-peptide > 300 pmol/L. While it is suggested that IDAA1C is an alternative to directly measuring c-peptide [5], it underestimates the proportion of patients who have a stimulated c-peptide > 200 pmol/L, especially in a paediatric population [6].

### 5.2. 2b Model-Estimated Average Plasma C-peptide Concentration

The model-Estimated Average Plasma C-peptide Concentration (CP_EST_) is a suggested clinical model which uses routinely measured clinical parameters from a single timepoint to estimate beta cell function. The model includes disease duration, body mass index, insulin dose, HbA1c, fasting plasma c-peptide and fasting plasma glucose to estimate the average plasma c-peptide result from an MMTT [120]. Favourable results are reported compared with the previously described IDAA1C (area under ROC 0.89, 95% CI 0.87, 0.92 vs. area under ROC 0.72, 95% CI 0.68, 0.76, respectively). This model has been validated as a potential substitute for simulated c-peptide testing, with a strong correlation between this model and the AUC of meal-stimulated c-peptide (Spearman’s R = 0.911, 95% CI 0.892, 0.926). This is favourable when compared to the correlation between the previously described IDAA1C and the AUC of meal-stimulated c-peptide (Spearman’s R = −0.555, 95% CI −0.619, −0.484) [121]. This model has therefore been suggested as an alternative to stimulated c-peptide testing in future interventional trials [121].

### 5.3. 2c Model-Estimated Stimulated Peak C-peptide Concentration

This third clinical model to estimate beta cell reserve includes age, body mass index, gender, HbA1c and insulin dose to predict 90 min stimulated c-peptide results [94]. Instead of including c-peptide measurement, which is not routinely measured in children with T1D, this model includes variables which contribute to insulin sensitivity as a surrogate for a real-time c-peptide levels. This acknowledges that c-peptide measurement reflects both the secretory function of beta cells and insulin sensitivity. This model is also reported to have a better predictive value than the IDAA1C model in estimating the 90 min stimulated c-peptide result (adjusted R^2^ = 0.63, *p* < 0.0001 vs. R^2^ = 0.37, *p* < 0.0001, respectively). When validated in a larger clinical cohort, the model was not as accurate in predicting measured c-peptide levels. When estimated and stimulated 90 min c-peptide levels obtained at 6 months and 12 months post diagnosis were compared, R^2^ values were 0.36 and 0.37, respectively. When compared to measured c-peptide levels, this model underestimates higher c-peptide levels and overestimates lower levels. The aim of this model is not to replace stimulated c-peptide tests in clinical trials, but to offer a practical approach to estimating beta cell reserve in clinical situations [94].

## 6. Approach 3: Other Biomarkers

### 6.1. 3a Proinsulin/C-peptide Ratio

C-peptide and insulin are secreted in equal molar amounts following cleavage of proinsulin. The proinsulin/c-peptide ratio (PI:C) has been used as a biomarker of beta cell stress [122,123]. Fasting and MMTT-stimulated PI:C increased from baseline measurement early in T1D diagnosis to measurements at 12 months; demonstrating increasing beta cell stress. One year after diagnosis, those with an IDAA1C > 9 (i.e., considered not be in remission) had a higher PI:C. Patients who had a higher PI:C at baseline were shown to have a greater decline in their c-peptide over the first year of disease [123]. Although these results may signify beta cell stress, PI:C has not been used to define a partial clinical remission to date.

### 6.2. 3b Cytokines

Cytokines have been investigated for their potential use as biomarkers of beta cell function. Tumour necrosis factor (TNF)-α, interleukin (IL)-2 and IL-6 have been found to be inversely correlated with stimulated c-peptide levels. TNF-α and IL-10 levels measured early in the disease process may also correlate with stimulated c-peptide levels at 6 months, and have therefore been suggested as possible biomarkers of beta cell reserve, which can be measured in fasting plasma samples [20]. IL-8 has also been suggested as a potential marker of clinical remission in patients with new-onset T1D [59].

Adipokines have also been investigated for their use in quantifying beta cell reserve. Serum leptin and resistin levels positively correlate with fasting c-peptide and MMTT-simulated c-peptide levels [124]. To date, prospective studies have not utilised cytokines as the primary measure of beta cell function.

### 6.3. 3c MicroRNA

MicroRNA (miR)-204 is another potential biomarker of beta cell function. MiR-204 is be released from apoptosing beta cells and plays a role in insulin production and secretion [125]. Levels are not increased in the serum of patients with T2D or other autoimmune diseases, suggesting that this may be a marker of beta cell decline in this condition. Serum levels of miR-204 inversely correlate with MMTT-stimulated c-peptide AUC [125]. Similar to other potential biomarkers, it has not yet been used in a study to define the honeymoon period.

## 7. Limitations of Beta Cell Reserve Assessment

### 7.1. Various Cut off Values

While variable methods to assess beta cell reserve have been described, there are differences noted in their interpretation across each method studied. Mixed meal-stimulated c-peptide thresholds of ≥300 pmol/L at 120 min, >300 pmol/L at 90 min, or AUC ≥ 200 pmol/L have been used to define patients with residual beta cell function. Similarly, some studies using IDAA1C described a value of ≤9 to define the honeymoon period, whereas others used <9, potentially classifying patients differently across studies. Studies using TDD and Hba1c as a marker of remission also use widely variable cut-offs to define partial remission in their population (Table 1).

### 7.2. Various Immunoassays

In addition to variation in reported thresholds, there are differences in assays used in measuring c-peptide concentrations across studies. These include chemiluminescence immunoassays and fluoroimmunoassays. Despite the use of different assays, some studies have used the same c-peptide threshold to define residual beta cell function (Table 1). This may be a limitation, as differences in reported measurements have been described between assays [126]. Assay standardisation should be considered in future clinical trials describing partial clinical remission.

### 7.3. Insulin Sensitivity

Insulin resistance in T1D contributes to beta-cell decline [127,128,129] and varies between individuals according to factors including age, sex, puberty and body mass index. Directly measuring c-peptide levels may provide information on both the secretory ability of beta cells and insulin sensitivity of the individual [94], although it is argued that serum c-peptide levels alone do not accurately reflect insulin resistance. If this is the case, defining a partial clinical remission in T1D by measuring stimulated c-peptide alone is insufficient and measures of insulin resistance would be required [128]. In one longitudinal study, the IDAA1C was compared to stimulated c-peptide levels in children with T1D [93]. Partial clinical remission was defined as IDAA1C ≤ 9, and significant beta cell function as stimulated c-peptide > 300 pmol/L. More than a year after T1D diagnosis, almost 55% of the study cohort who had a stimulated c-peptide > 300 pmol/L were not in partial remission as defined by their IDAA1C. This group of patients were reported to have a significantly lower insulin sensitivity (*p* < 0.001), as measured by an insulin sensitivity score developed in 2011 to estimate insulin sensitivity [130].

A large cohort study found that children diagnosed with T1D at age 5 years or younger were significantly less likely to have a partial remission period compared to children who were older at the time they were diagnosed [70]. They described this finding as biphasic. Although early in the study it appeared that younger children had a more aggressive disease course with lower probability of remission, a greater proportion of this group of younger patients were in remission compared to their older peers at the end of the 6-year observation. Their explanation for this finding was linked to insulin sensitivity. Older children may have had a less aggressive disease course, but fewer of them met the IDAA1C criteria for remission because insulin sensitivity is reduced with puberty. Children diagnosed at a younger age were more likely to still be prepubertal at the end of the 6-year study.

### 7.4. Patient Population

Some methods of measuring beta cell reserve are not as practical for a paediatric population as in adults. The MMTT can take up to four hours and involves the placement of an intravenous cannula for blood sampling, which can be problematic in the paediatric population [110]. Similarly, nausea has been described as a common adverse effect for paediatric patients undergoing glucagon stimulation tests, compared with older patients [111]. These practical issues may also complicate translation of these research tests to clinical practice.

## 8. Conclusions

The emergence of approved therapies that may modify the rate of beta cell decline in T1D places a renewed focus on the need to quantify this trajectory. In this review, we have highlighted the various models that have been used to describe beta cell reserve and provided comparative data to guide their use. AUC c-peptide and peak-stimulated c-peptide levels are the most reproducible measures of beta cell function, and limitations of clinical models should be considered when used.

The definition of partial remission varies significantly between studies using the same modality to assess beta cell reserve, limiting comparisons between results across studies. Clinical models that do not necessitate prolonged, expensive testing are more practical in clinical settings where clinicians want to establish if patients are in remission or not, and are used most frequently at present. However, these approaches do not correlate closely with stimulated c-peptide measurement [120,121].

Future directions may include the use of continuous glucose monitor (CGM) data to highlight patterns associated with the remission. The utility of IDAA1C may be limited by the duration of glycaemic control reflected by HbA1c measurement. However, real-time CGM data combined with insulin doses and glycaemic variation may reflect active beta cell reserve more accurately [56,67]. Biomarkers, especially those related to beta cell stress, may also have a role in assessing effects of medical interventions to preserve beta cell function.

This review highlights the wide variation in defining partial remission in T1D. With treatments such as teplizumab now approved to protect beta cells, it is essential that an agreed definition of remission is used. This would ensure the reported efficacy of potential treatments is based on a standardised measurement.

## Figures and Tables

**Table 1:** Methodologies used in assessing beta cell reserve, and thresholds used to define residual beta cell function in included studies. HbA1c = haemoglobin A1c; IDAA1c = insulin dose-adjusted HbA1c; MMTT = mixed meal tolerance test; TDD = total daily dose. * Denotes the use of chemiluminescence immunoassay. ^ Denotes the use of fluoroimmunoassay.

Method	Threshold to Define Residual Beta Cell Function	Author
MMTT-Stimulated C-Peptide	≥300 pmol/L at 120 min	Li X * [15], Zhong T * [16], Chen Y * [17]
	>300 pmol/L at 90 min	Mortensen HB ^ [5] Max Anderson ML ^ [18], Madsen JOB * [19], Overgaard AJ * [20]
Insulin TDD and HbA1c	<0.5 U/kg/d, HbA1c < 8%	Kara O [21] Bowden SA [22]
	≤0.5 U/kg/d, HbA1c ≤ 7.5%	Verrijn SAA [23] Ortqvist E [24], Meng X [25]
	<0.5 U/kg/d, HbA1c < 7.5%	Nordwall M [26]
	≤0.5 U/kg/d, HbA1c < 7.5%	Araujo DB [27]
	<0.5 U/kg/d, HbA1c < 7%	Pyziak A [28] Jamiolkowska-Sztabkowska [29,30,31], Souza L [32], Villalba A [33]
	≤0.5 U/kg/d, HbA1c < 7%	Wang Y [34] Pediatric Diabetes Consortium [35] Dost A [36]
	<0.5 U/kg/d, HbA1c ≤ 7%	Chobot A [37]
	<0.5 U/kg/d, HbA1c ≤ 6%	Abdul-Rasoul M [38]
	≤0.4 U/kg/d, HbA1c < 7%	Humphreys A [39]
	≤0.38 U/kg/d, HbA1c < 7.5%	Schloot NC [40]
	<0.1 U/kg/d, “normal HbA1c” for >3 weeks	Barone R [41]
	<10 units/day, “normal metabolic state”	Kamado K [42]
	<0.3 U/kg/d, “normal” HbA1c for at least 10 days	Bonfanti R [43]
	<0.5 U/kg/d, HbA1c ≤ 7.9%, preprandial blood glucose ≤ 8mM	Cook JJ [44]
Insulin TDD	<0.5 U/kg/d	Bober E [45], Sanda S [46] Kordonouri O [47] Glisic-Milosavljevic S [48] Muhammad BJ [49], Cadario F [50]
	≤0.49 U/kg/d	Meng X [25]
	<0.3 U/kg/d	Lundberg RL [51] Jamiolkowska-Sztabkowska [31] Nwosu BU [52]
	<0.25 U/kg/d	Feutren G [53]
	≤50% of dose at time of discharge	Glisic-Milosavljevic S [48] Agner T [54]
Clinical Model-IDAA1C	<9	Blair JC [55], Polle OG [56], Addala A [57], Kingery SE [58], Villalba A [33] Pyziak-Skupien A [59] Cadario F [60] Casas R [61] Fonolleda M [2]
	≤9	Mortensen HB [5], Gomez-Munoz L [62,63], Nwosu BU [52,64,65,66], Yesiltepe-Mutlu [67], Chiavaroli V [68], Pecheur A [69], Nagl K [70], Moya R [71], Narsale A [72], Kaas A [73], Bechi Genzano C [74], Lundgren M [75], Klocperk A [76], Lawes T [77], Pinckney A [78], Cengiz E [79], Neylon OM [80], Moosavi M [81], McGill DE [82], Max Andersen ML [18], Madsen JOB [19], Zhong T [16], Li X [15], Neuman V [83], Quattrin T [84], Cabrera SM [85] Pyziak A [28], Lundberg RL [51] Passanisi S [86], Marino KR [87] Camilo DS [88], Nielens N [89] Redondo MJ [90] Franceschi R [91] Cimbek EA [92], Mork FCB [93]
Clinical Model-Glycemic Target-Adjusted HbA1c	≤4.5	Nielens N [89]
Clinical Model-Estimated C-Peptide Model	Clinical Model to estimate 90 min stimulated c peptide	Buchanan K [94]
Other	Insulin TDD < 0.5 U/kg/d and minimal/no glycosuria	Hocking MD [95]
	Insulin TDD < 0.3 U/kg/d and “proper glycaemic control” and c-peptide > 0.5 ng/ml	Jamiolkowska-Sztabkowska [31]
	Insulin TDD ≤ 0.3 U/kg/d and HbA1c < 7% and a random serum c-peptide > 0.5 ng/ml	Pilacinski S [96]
	Insulin TDD < 0.5 U/kg/d and no glycosuria and “detectable c-peptide”	Vetter U [97]
	Insulin TDD < 0.5 U/kg/d and absent/minimal glycosuria for >4 weeks and period of no clinical symptoms	Al Rashed AM [98]
	Insulin TDD < 0.5 U/kg/d and minimal/no glycosuria for ≥1 month	Ludvigsson J [99,100]

## Abbreviations

AUC	Area Under the Curve
CGM	Continuous Glucose Monitor
CP_EST_	Model-Estimated Average Plasma C-peptide Concentration
HbA1c	glycosylated haemoglobin A1c
IDAA1C	Insulin Dose-adjusted A1C
MMTT	Mixed Meal Tolerance Test
PI:C	Proinsulin/C-peptide ratio
TNF	Tumour Necrosis Factor
IL	Interleukin
T1D	Type 1 Diabetes
TDD	Total Daily Dose
UCCR	Urinary C-Peptide/Creatinine Ratio
